# Patients’ use of mHealth in their obesity treatment: a Swedish descriptive observational study

**DOI:** 10.1136/bmjph-2025-004035

**Published:** 2025-10-13

**Authors:** Kajsa Cederblad, Madeleine Blusi, Stephanie E Bonn, Anne Christenson

**Affiliations:** 1 Center for Obesity, Academic Specialist Center, Stockholm Health Services, Stockholm, Sweden; 2 Department of Nursing, Umeå University, Umeå, Sweden; 3 Department of Medicine Solna, Clinical Epidemiology Division, Karolinska Institutet, Stockholm, Sweden; 4 Department of Medicine Huddinge, Karolinska Institutet, Stockholm, Sweden

**Keywords:** Digital Health, Obesity, Health Services Accessibility, Adolescent, Sociodemographic Factors

## Abstract

**Introduction:**

Successful obesity treatment involves frequent follow-up contacts, but the use of mHealth contacts has not been evaluated. We aimed to investigate patients’ use of an mHealth chat in their obesity treatment.

**Methods:**

In this descriptive observational study, we included 492 patients, 16 years or older, from a specialist obesity treatment clinic in Sweden. We assessed chat usage during their first 6 months of treatment, and compared subgroups, considering factors such as age, sex, body mass index (BMI), socioeconomic status, prescribed weight management medications and digital welcome letter.

**Results:**

Participants had a mean BMI of 42.5 kg/m^2 ^and a mean age of 30.5 years, 73.4% were women. Over 90% of participants had low socioeconomic status and 23.8% were prescribed weight management medications. Almost all participants (90%) used the chat function during their first 6 months of treatment. Younger participants, those prescribed weight management medications, and those who received a digital welcome letter before admission were the most active users. Socioeconomic status or sex did not appear to be associated with chat activity.

**Conclusion:**

Integrating mHealth solutions, including chat function, into obesity treatment programmes can be a way to maintain patient contact, also for patients with low socioeconomic status. Healthcare provider-initiated digital interactions may encourage greater patient engagement.

**WHAT IS ALREADY KNOWN ON THIS TOPIC**

**WHAT THIS STUDY ADDS**

- Sex or socioeconomic status was not associated with chat usage activity.

**HOW THIS STUDY MIGHT AFFECT RESEARCH, PRACTICE OR POLICY**

- Healthcare provider-initiated digital interactions may increase patient engagement, regardless of patients’ socioeconomic status.

## Introduction

 The prevalence of obesity has reached epidemic levels globally, causing health issues like cardiovascular diseases, type 2 diabetes and cancer.^1^ Approximately 5 million deaths annually are linked to overweight or obesity.^2^ Beyond physical consequences, obesity leads to negative psychosocial effects and stigmatisation.^3^ Globally, adult obesity has more than doubled since 1990, while adolescent obesity has quadrupled during the same time.^2^ In 2022, 2.5 billion adults were overweight, with 890 million living with obesity.^2^ In light of this, more effective and accessible obesity treatment is needed.

Successful obesity treatment includes frequent contact with healthcare staff during the first year.^4^ Today, digital health, encompassing various aspects of electronic health including mobile health (mHealth), is broadly available. It offers advantages such as asynchronous communication and improved access to care in remote areas.^5^ Furthermore, interactive health applications (apps), through which healthcare professionals can send health-related text messages, have demonstrated positive effects on patients' behaviours, including increased physical activity and improved diet.^6^ Messages can be timed to suit individual needs,^7^ and communication via text allows for thoughtful responses, providing reviewable messages for clarity and encouragement.^8 9^

Access to various communication channels to health professionals may increase patient’s ability to seek, find, understand and use information and services to informed health-related decisions and actions for themselves and others. When information is derived from electronic sources, it is known as eHealth literacy.^10 11^ Low health literacy is linked to higher rates of hospitalisation and mortality,^12^ as well as higher body mass index (BMI), possibly via a poorer understanding of food labels and underestimation of portion sizes.^13^ Patients with chronic conditions and low health literacy often show less confidence in making lifestyle changes, have less proactive health behaviours and avoid adequate healthcare.^14^ This may complicate specialist obesity treatment, as many patients have multiple chronic conditions.

Although helpful in other settings, the use of an mHealth chat function remains understudied in specialist obesity care. We aimed to investigate patients’ use of a mHealth chat at a specialist obesity treatment centre, focusing on differences by sex, age, BMI, use of obesity management medication and socioeconomic background in patients. We also wanted to evaluate if the digital welcome letter, sent to young adults prior to admission, affected chat usage during treatment.

## Methods

### Study design and setting

This was a descriptive observational study using data from the chat function in a healthcare app combined with information obtained from medical records.

The app is available to all 2.5 million residents in the region at no cost for the individual, and the chat function allows patients and healthcare professionals to communicate via text, and to send photos or documents. Since 2019, a specialist obesity treatment centre is using the chat function in the mHealth app as a complement to regular care. This study was conducted from September 2021 to May 2024 at the obesity treatment centre which admits patients aged 16–70 years. The centre offers weight reduction treatment, provided by a multiprofessional healthcare team. The lifestyle treatment can be complemented with obesity management medication after individual medical evaluation by a physician. During the study period, three approved obesity management medications could be used in Sweden, alongside lifestyle treatments: naltrexone/bupropion, orlistat and glucagon-like peptide-1 agonists.^15^

### Patient involvement

The chat function was developed in close communication with patients before this study, to take their experiences and preferences into account. Though patients were not involved in choosing the methods or outcome measures for this study, the research questions originate from this patient-healthcare provider discussions. Patients will benefit in the dissemination of the results, as all new knowledge and suggestions for improvements can be directly put into practice at the clinic.

### Recruitment

Study participants were recruited consecutively at the obesity treatment centre during the study period. Patients with intellectual disabilities, those requiring an interpreter, or those with protected identities, or who were undergoing gender-affirming treatment, were exempt, as you cannot access the chat function without a valid personal identification number, and individuals with protected identity are advised not to use the app. All eligible patients admitted to the centre during this period, n≈800, were provided study information in connection with their admission consultation, that is, their first visit to the centre. In total, 514 patients consented to participate.

### Consent to participate

Participants received verbal and written information about the study, including that their treatment would not be affected if they chose not to participate. Patients willing to partake signed an informed consent. According to the Ethics Review Act (2003:460), individuals aged 15–18 years can provide informed consent for research participation without requiring parental consent, if they understand its implications.

### The mHealth application

The app evaluated in this study includes a chat function which enables patients and healthcare professionals to exchange texts, images and documents. It is asynchronous and available during all hours of the day. Healthcare professionals can also send standardised templates, such as reminders for blood tests, and are able to see whether patients have read their messages. Healthcare professionals review and respond to patients’ text messages during the daytime on weekdays. At the participating clinic, patients aged 16–25 years were encouraged to download the app prior to admission to receive a digital welcome letter containing details about their admission appointment. This initiative was part of a proactive effort at the clinic to engage younger adults. Patients older than 25 years were not provided such instructions.

### Data collection

Chat activity was collected via the app from each participant for 6 months from enrolment. To capture gradations in patient engagement, we created a categorical variable with five levels, defining participants' text message activity (TMA) in the app. The definitions were based on earlier observations of chat behaviour and temporal patterns, and the thresholds and assignment rules were researcher-determined.

Participants were classified into one of five categories that described the activity level from enrolment and 6 months onwards (table 1). *Category 1*: The most active participants who initiated text messages without prior digital contact, except for the digital welcome letter sent to 16–25 year-olds at admission. *Category 2*: Patients who had previous contact with a healthcare professional but later initiated new contact themselves. A chat was considered self-initiated if it occurred more than 2 days after a previous message or if a new topic was introduced; the 2-day interval was chosen a priori by the researcher as a reasonable threshold based on observed communication patterns. *Category 3*: Patients who did not initiate any chat communication but responded to messages from their healthcare professional. *Category 4*: Patients who read, but did not respond to, messages from their healthcare professional. *Category 5*: Participants who neither received nor read, nor initiated any messages.

**Table 1:** Text message activity (TMA) categorisation

Category 1	Patient has initiated TMA without previous digital contact with HCP
Category 2	Patient has initiated TMA*
Category 3	Patient has answered TMA initiated from HCP
Category 4	Patient has read TMA but not replied
Category 5	Patient has not used TMA, that is, neither initiated nor received or read message from HCP

* Patient-initiated chat is defined as self-initiated if it occurs more than.2 days after the previous message or if the topic is clearly different from previous message exchanges within an open thread.

HCP, healthcare professional.

Background data of date of birth, biological sex, zip code of residence, height and weight at enrolment, and information on prescribed obesity management medications were obtained from medical records. Zip codes were used to estimate socioeconomic levels, using a public website with income data for residential areas, sourced from Statistics Sweden in June 2023. Socioeconomic status was defined as either high or low, depending on whether participants resided in an area with a monthly income above or below the Swedish average.

### Statistical analysis

Descriptive statistics characterised the study population, with mean and SD for continuous variables, and number and percentage (n, %) for categorical variables. Analysis of variance compared the means of age, weight and BMI across TMA categories.^16^ Pearson’s χ^2^ test assessed differences in the distribution of categorical variables between TMA categories, including sex, age groups, BMI classes, obesity management medications, socioeconomic backgrounds and if participants had received a digital welcome letter or not. The significance level for all tests was set at 0.05. Statistical calculations were performed using STATA 17.

## Results

At the time for analysis, 22 participants were excluded due to missing chat data. They were individuals who received protected identities (n=4), those who signed consent but never commenced treatment during the study period (n=12) and participants who changed personal identification numbers (n=6), such as individuals undergoing gender-affirming treatment, during the study. Characteristics of the 492 study participants included in the analysis are presented in table 2. Most participants (90.0%, n=443) had used the chat in some way during their first 6 months of treatment, that is, belonged to TMA categories 1–4. Most participants (73.4%, n=361) were women. The mean age was 30.5 (SD 14.4) years, with 62.0% (n=305) of participants aged 16–25 years. The mean BMI was 42.5 (SD 6.6) kg/m^2^, and 60.2% (n=296) of participants had a BMI≥40 kg/m^2^. Obesity management medications had been prescribed to 23.8% (n=117) of participants, of whom 10 had been prescribed more than one medication. Most participants had a low socioeconomic status (90.3%, n=438).

**Table 2:** Baseline characteristics of study participants divided by text message activity (TMA) category, where participants in category 1 were the most active and participants in category 5 were the least active

Variables		TMA category					P value*
	All (n=492)	1 (n=49)	2 (n=138)	3 (n=195)	4 (n=61)	5 (n=49)	
	Mean (SD)	Mean (SD)	Mean (SD)	Mean (SD)	Mean (SD)	Mean (SD)	
Age, years	30.5 (14.4)	29.4 (11.1)	29.2 (12.9)	29.9 (14.3)	30.3 (15.9)	38.1 (17.9)	0.004
Weight, kg	123.5 (24.7)	129 (32.3)	121.7 (20.5)	122.6 (24.5)	122.1 (24)	128.4 (27.7)	0.23
BMI, kg/m^2^	42.5 (6.6)	43.8 (9.7)	42.2 (5.8)	42.2 (6.6)	42.2 (5.4)	43.9 (6.8)	0.28
	**n (%**)	**n (%**)	**n (%**)	**n (%**)	**n (%**)	**n (%**)	
Sex							0.051
Male	131 (26.6)	10 (20.4)	26 (18.8)	59 (30.3)	22 (36.1)	14 (28.6)	
Female	361 (73.4)	39 (79.6)	112 (81.2)	136 (69.7)	39 (63.9)	35 (71.4)	
Age, years							0.038
16–25.9	305 (62.0)	31 (63.3)	92 (66.7)	124 (63.6)	39 (63.9)	19 (38.8)	
26.0–44.9	84 (17.1)	11 (22.5)	20 (14.5)	31 (15.9)	11 (18)	11 (22.5)	
≥45	103 (20.9)	7 (14.3)	26 (18.8)	40 (20.5)	11 (18)	19 (38.8)	
BMI, kg/m^2^							0.41
<35.0	36 (7.3)	4 (8.2)	7 (5.1)	19 (9.7)	2 (3.3)	4 (8.2)	
35.0–39.9	160 (32.5)	15 (30.6)	50 (36.2)	64 (32.8)	21 (34.4)	10 (20.4)	
≥40	296 (60.2)	30 (61.2)	81 (58.7)	112 (57.4)	38 (62.3)	35 (71.4)	
Obesity management medication							
Any	117 (23.8)	11 (22.5)	55 (39.9)	44 (22.6)	6 (9.8)	1 (2)	<0.001
SES status†							
Low	438 (90.3)	43 (89.6)	117 (86.7)	175 (90.7)	55 (91.7)	48 (98)	0.24
High	47 (9.7)	5 (10.4)	18 (13.3)	18 (9.3)	5 (8.3)	1 (2)	
Digital welcome letter before enrolment							<0.001
Yes	237 (48.2)	25 (51.0)	69 (50.0)	104 (53.3)	33 (54.1)	6 (12.24)	
No	255 (51.8)	24 (49.0)	69 (50.0)	91 (46.7)	28 (45.9)	43 (87.8)	

* Analysis of variance (ANOVA) for continuous variables and χ2 test for categorical variables.

† Missing data n=7, living in an area with a mean income below the average Swedish income according to Statistic Sweden (June 2023).

The proportion of women was slightly higher in the most active TMA category 1 (79.6%, n=39) and 2 (81.2%, n=112), compared with category 3 (69.7%, n=136), 4 (63.9%, n=39) and 5 (71.4%, n=35), but there was no statistically significant difference in distribution of TMA categories between women and men. A statistically significant difference in age was observed between TMA categories (p=0.004), with participants in higher (less active) categories being older. There were no differences in weight, BMI or SES between TMA categories. A statistically significant difference was detected regarding participants who had a prescribed obesity management medication (p=0.001) as they were more active in the chat compared with participants with no obesity management medication. A significant difference in TMA category distribution (p=0.001) was observed between individuals who received a welcome letter before their admission appointment and those who did not. Very few participants in the least active category (category 5) had received a welcome letter.

## Discussion

Almost all participants used the mHealth chat function during their first 6 months of obesity treatment to interact and communicate with healthcare professionals. A significantly higher chat activity was seen among patients who had received a previous text message from healthcare professionals. Our results indicate that healthcare provider-initiated interactions may be associated with patients’ use of mHealth. Introducing patients to a communication channel where they can influence the timing and frequency of healthcare contacts may also facilitate patient-centred care.

Our findings showing that younger participants used the chat more often align with previous research. Older age has been shown to negatively influence the acceptance and intention to use mHealth solutions.^17^ Younger individuals are in general more likely to have digital doctor visits compared with older individuals,^18^ and text messages have been shown to be especially suitable for adolescents.^19^ However, as only our younger participants received a welcome letter, we cannot determine whether it was the participants' age or the letter they received that was associated with their level of chat activity. Additional potential factors that may be associated with the level of chat activity, but that were not assessed in this study, include participants’ educational level, general differences in engagement with obesity treatment and variations in e-health literacy.

Approximately 10% of our participants did not use the chat function during their first 6 months of obesity treatment. Exploring individual reasons is beyond the scope of this study, but it is possible that some participants did not require the additional contact. Furthermore, participants who were not encouraged to download the app may have been unaware of its existence. However, according to data from the app’s backend system, approximately 85% of all residents aged 18 or older had downloaded and activated the app. It is also possible that some participants were less inclined to use the chat, knowing that their messages may be reviewed by researchers. In addition, we only examined their first 6 months of treatment and with time, more participants may have used the chat. If so, it would mean that an even higher proportion of participants may have been willing to use the chat function outside study settings.

In our study, all participants were individuals living with the chronic disease obesity, and over 90% had a low SES. Most participants (382 out of 492) had actively interacted with health professionals by sending a message via the mHealth app at some point. This was unexpected as previous studies indicate that while most patients read their electronic messages, socioeconomically disadvantaged groups use web portals less frequently.^20^

With rising obesity rates, it is crucial to improve treatment interventions as well as their availability.^21^ Obesity treatment should be personalised and consider the various needs in a very heterogenic patient group.^22^ Earlier studies have shown that interactive apps can increase health literacy in patients,^23 24^ and that it is important to take health literacy into consideration when designing health apps.^24^ Future research should further examine the relationship between SES and health literacy, and how these factors may influence engagement with digital health interventions. Furthermore, it is important that the sender considers factors such as age, education, sex, cultural and socio-economic background,^8^ as for example young adults dislike authoritarian tones.^19^ Also, there is a fine line between being perceived as supportive or intrusive, and therefore healthcare professionals should consider at what stage the patient is in his or her treatment journey.^25^ The message tone and wording were not the focus of this study but could be further explored in future research. Perhaps providing easy access mHealth apps for digital communication such as text messaging, with trusted, well-known healthcare professionals, can become an important component in the treatment toolbox for individualised obesity treatment.

A limitation to our study was the exclusion of 22 participants due to missing data. The exclusion of these participants may have introduced bias, as they would likely have been less active chat users.

## Conclusion

Low SES may not be a hindering factor for chat communication in obesity treatment. Integrating an mHealth solution, including a chat function, can be a way to maintain patient contact and to follow-up obesity management medication treatment. Proactive initiation of chat contact from healthcare professionals may increase mHealth usage among participants.

## Data Availability

Data are available upon reasonable request.
